# Moving forward through the in silico modeling of multiple sclerosis: Treatment layer implementation and validation

**DOI:** 10.1016/j.csbj.2023.05.020

**Published:** 2023-05-20

**Authors:** Avisa Maleki, Elena Crispino, Serena Anna Italia, Valentina Di Salvatore, Maria Assunta Chiacchio, Fianne Sips, Roberta Bursi, Giulia Russo, Davide Maimone, Francesco Pappalardo

**Affiliations:** a Department of Mathematics and Computer Science, University of Catania, Viale Andrea Doria 6, Catania 95125, Italy; b Department of Biomedical and Biotechnological Sciences, University of Catania, Via Santa Sofia 97, Catania 95125, Italy; c Department of Drug and Health Sciences, University of Catania, Viale Andrea Doria 6, Catania 95125, Italy; d InSilicoTrials Technologies BV, 's Hertogenbosch, the Netherlands; e Mimesis SRL, Catania, Italy; f Centro Sclerosi Multipla, UOC Neurologia, ARNAS Garibaldi, P.zza S. Maria di Gesù, Catania 95124, Italy

**Keywords:** Multiple sclerosis, Clinical decision support systems, Digital twins, In silico trials, Validation, Modelling and simulation

## Abstract

Multiple sclerosis is an autoimmune inflammatory disease that affects the central nervous system through chronic demyelination and loss of oligodendrocytes. Since the relapsing-remitting form is the most prevalent, relapse-reducing therapies are a primary choice for specialists. Universal Immune System Simulator is an agent-based model that simulates the human immune system dynamics under physiological conditions and during several diseases, including multiple sclerosis. In this work, we extended the UISS-MS disease layer by adding two new treatments, i.e., cladribine and ocrelizumab, to show that UISS-MS can be potentially used to predict the effects of any existing or newly designed treatment against multiple sclerosis. To retrospectively validate UISS-MS with ocrelizumab and cladribine, we extracted the clinical and MRI data from patients included in two clinical trials, thus creating specific cohorts of digital patients for predicting and validating the effects of the considered drugs. The obtained results mirror those of the clinical trials, demonstrating that UISS-MS can correctly simulate the mechanisms of action and outcomes of the treatments. The successful retrospective validation concurred to confirm that UISS-MS can be considered a digital twin solution to be used as a support system to inform clinical decisions and predict disease course and therapeutic response at a single patient level.

## Graphical Abstract

**Figure:**
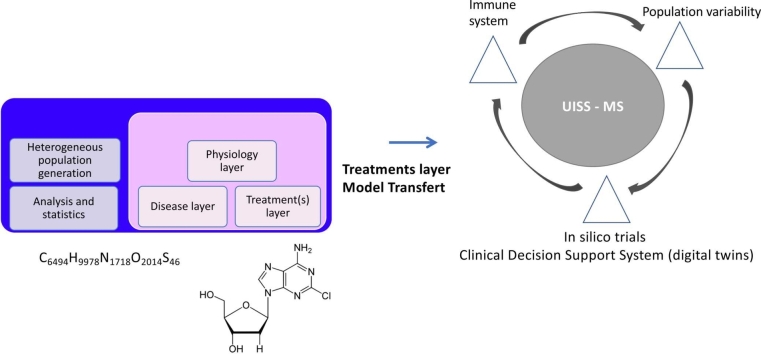

## 1 Introduction

Multiple Sclerosis (MS) is a chronic inflammatory demyelinating disease of the Central Nervous System (CNS) [1]. Epidemiological data reveal that genetic and environmental factors are pivotal in MS development. In particular, lack of vitamin D, Epstein–Barr virus (EBV) infection, daily stress, obesity, and smoking are considered the main factors that could contribute to the development of MS [2], [3]. Sensory symptoms are considerable in patients with MS, then pain syndromes, respiratory symptoms, fatigue, vertigo, visual disturbance, sensorimotor defects, stiffness of the muscles, and paralysis are significantly frequent in MS patients [4].

The course of MS is heterogeneous, and according to the US National Multiple Sclerosis Society (NMSS) Advisory Committee on Clinical Trials in Multiple Sclerosis in 1996 [5], four MS clinical courses could be identified: relapsing-remitting MS (RRMS), primary progressive MS (PPMS), secondary progressive MS (SPMS), and progressive relapsing MS (PRMS). RRMS is the most common clinical phenotype at onset, which may eventually turn into SPMS, depending on several factors such as age, disease duration, and treatment [6]. Approximately 15 % of patients are diagnosed with PPMS at onset and exhibit relentless worsening of neurological status without remission [7]. PPMS is characterized by reduced inflammation compared to relapsing forms, lower lesion activity on MRI, and reduced inflammation within the CNS (i.e., neutrophils, monocytes, lymphocytes) [8]. Therefore, the course descriptions were sometimes amalgamated into relapsing (RMS., including RR, SP, and PR) and progressive (PMS, including PP, SP, and PR) forms, considering the main distinction. However, it was never adequately defined whether the subject's disease was mostly relapsing or progressive [5].

MS is hypothesized as a multifactorial and autoimmune disease, and its etiology is still unclear. However, evidence indicates that T and B cells are essential in generating a systemic autoimmune response that eventually invades the CNS and produces inflammation and demyelination [9], [10]. There are several approved drugs for MS patients, including natalizumab, ocrelizumab, alemtuzumab, interferon beta (IFNβ), glatiramer acetate (GA), mitoxantrone, dimethyl fumarate, fingolimod, teriflunomide, and cladribine.

GA is an immunomodulating amino acid copolymer that received FDA approval in 1996 [11]. T cell activation and generation of Th2 cells are part of its mode of action. Th2 cells can act as immunomodulators by promoting the production of anti-inflammatory cytokines such as interleukin (IL)− 4, IL-10, and TGF- β [12].

The signaling pathways for sphingosine 1-phosphate (S1P) have several significant roles. Due to their role in controlling lymphocyte trafficking, brain and heart function, vascular permeability, and vascular and bronchial tone, S1P receptors (S1PRs) have been recommended as therapeutic target for several disorders. For example, S1PR modulators are now solely approved for treating multiple sclerosis. Fingolimod, siponimod, ozanimod, and ponesimod are four S1PR modulators that have regulatory approval for multiple sclerosis. The primary mechanism of action of S1PR modulators in MS is the binding of S1PR subtype 1 on lymphocytes, which results in the internalization of the receptor and loss of responsiveness to the S1P gradient, which drives lymphocyte egress from lymph nodes. Thus, the decrease in circulating lymphocytes probably inhibits the recruitment of inflammatory cells into the CNS [13].

B cells have a leading role in the pathogenesis of MS through the involvement in the inflammatory T cell activation, secretion of pro-inflammatory cytokines, and production of myelin-targeting autoantibodies. Recently, the B cells depletion by anti-CD20 monoclonal antibodies was demonstrated effective for treating RRMS and PPMS [14]. Among these antibodies, rituximab, ocrelizumab, and ofatumumab have been tested in multiple sclerosis [15]. Ocrelizumab is a humanized recombinant antibody intended to target only cells with the B lymphocyte antigen CD20 on their surface [16].

Cladribine is a chlorinated analogue of deoxyadenosine that, after oral administration, exerts immunosuppressive activity by rapidly and sustainably depleting CD4 + and CD8 + T cells as well as rapid, though more transient, effects on CD19 + B cells [17], [18]. Moreover, cladribine plays a significant role in treating RRMS, and its efficacy was demonstrated in clinical trials [19].

Universal Immune System Simulator (UISS) is an agent-based model developed to simulate human immune system behavior in physiological or pathological conditions, such as infectious diseases, autoimmune diseases, and tumors. It considers cellular and molecular entities and can model different biological scenarios using a multi-layer approach (physiological, disease, and treatment based). In the last few years, several mechanistic models have been proposed to describe MS dynamics and the related immune system response [20]. In this context, we developed the disease layer on the UISS based on the most updated knowledge of MS physiopathology (UISS-MS). UISS-MS can process a large amount of essential information required to develop a disease profile at an individual level, helping to simulate the disease progression and select the best therapeutic option [21]. Within this aim, we used UISS to simulate the effects of several treatments both at individual and population levels. Specifically, in our previous study, we simulated and evaluated the effects of several drugs approved for MS disease, including teriflunomide, fingolimod, IFNβ− 1a, and natalizumab [22]. Here, we present the results obtained with two more treatment layers able to capture and simulate the mechanism of action of two drugs, ocrelizumab, and cladribine.

To retrospectively validate the performance of UISS-MS with ocrelizumab and cladribine, we considered two clinical trials from which we extracted the features of real patients [23], [24], [25]. In addition, we created a cohort of digital patients on which we predicted the efficacy and eventually verified the adverse effects of the considered drugs. With this new add-on, we expand the application field of UISS-MS as a mechanistic modeling and simulation platform to describe the main MS-modifying treatments. In particular, we prove that UISS-MS can potentially be used to predict the therapeutic value of most MS treatments and to provide digital twins to help neurologists in profiling patients and selecting the appropriate therapy.

## 2 Methods

The expression “*in silico trials*” refers to using personalized computer modeling and simulation in designing or assessing a drug therapy, a medical device, or a therapeutic intervention for treating or preventing a specific disease. In this field, UISS represents a mechanistic computational platform that simulates the human immune system to evaluate the effect of therapeutic or preventive strategies when used against different diseases. MS involves the main immune system features through a complex mechanism of action. UISS has already been used to simulate both MS course and treatment effects in heterogeneous populations of virtual RRMS patients [21]. The simulator is built on a state-of-the-art agent-based model which describes the RRMS patient in terms of *(i)* a complete, multifunctional description of the immune system, *(ii)* a disease-specific RRMS extension, and *(iii)* an easily extensible treatment module. The multifunctional base model has been deployed to describe a variety of immune-related pathologies and vaccines [26], [27], [28]. The model includes a detailed description of both innate and adaptive immune system, such as different cell types, key immune system processes, cytokines and chemokines signaling.

UISS uses a multi-layer approach to simulate the biological scenario of interest. Three main layers are considered:

1. physiology layer: it describes the physiological response of the human immune system to an entity considered “*non-self*” as well as to a “*self”* entity in the presence of immune system impairment;
2. disease layer: it represents the immune dynamics related to the mechanism of action and progression of a particular disease;
3. treatment layer: it depicts the effects a specific treatment used to control or prevent the disease has on the immune system.

Within the physiology layer, UISS implements anatomical compartments and the hallmarks of the human immune system (i.e., cells and molecules, immune system repertoire, molecular affinity, hematopoiesis with the generation of cells, cell maturation and thymus selection, aging, the memory of past infections, hyper-mutation of antibodies, bystander effect, cell activation and anergy, cell interaction and cooperation, antigen digestion and presentation).

In previous studies, we implemented the treatment layers for several drugs, including teriflunomide, fingolimod, IFNβ− 1a and natalizumab.

### 2.1 MS disease conceptual model

Creating a conceptual model is considered one of the critical steps in implementing a new disease layer. This also applies to developing any disease layer for the UISS platform, including MS. The MS conceptual model is a schematic representation of the entities involved in the disease, their interactions, and the immune system mechanism involved in the disease itself. Previously, we presented an overview of the ontology of a specific autoimmune interaction during MS dynamics [22]. The ontology concept formally defines and organizes the types, characteristics, and relationships among entities in a specific domain. Creating an ontology specifically tailored to MS contributes significantly to gaining a deeper understanding of the essential properties of the phenomenon [29].

The abovementioned ontology example illustrates the sequence of events where a previously activated cytotoxic T lymphocyte encounters its target, an oligodendrocyte, within the brain's white matter. The ontology defines the localization of each entity, which refers to the specific biological compartment they exist in (in this case, the brain), as well as their status, which pertains to the differentiation states an entity can possess (in this case, "activated" indicates being primed) [22].

However, in the current study, we improved the conceptual model of the MS disease layer, adding more details and releasing an updated version of its structure. This improvement is based on the most fundamental interactions (whether inhibitor or promoter) among entities that have an essential role in the pathophysiology of MS disease.

**Fig. 1:**
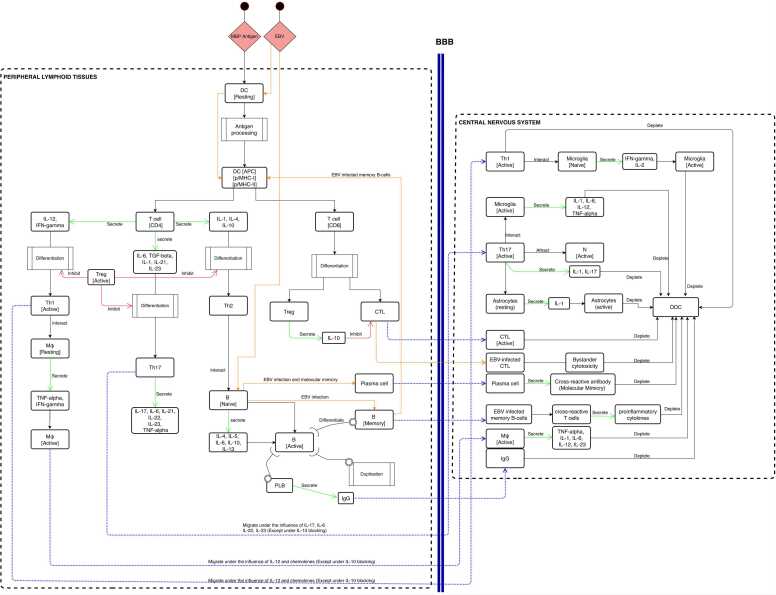
The MS – immune system interaction model. Conceptual description of the main immune system entities and interactions involved in the MS disease. The main two compartments, the peripheral lymphoid tissues and the central nervous system are depicted. The representation describes both cellular and humoral responses.

Fig. 1 reports the conceptual model of UISS-MS with the correlated details of its entities and interactions.

At the onset, the myelin basic protein (MBP) antigen is recognized by resting dendritic cells (DCs). Upon activation, the DC processes and presents antigenic peptides associated with major histocompatibility complex (MHC)-II to CD4 + T-cells and MHC-I to CD8 + T-cells. The differentiation of activated helper T cells into distinct subsets, such as Th1, Th17, and Th2, is regulated by specific cytokines. For example, the Th1 cells are characterized by the secretion of IL-12 and IFN-gamma (IFN-γ), while the secretion of IL-1, IL-6, IL-21, IL-23, and TGF-β characterizes Th17 cells. Also, Th2 cells are characterized by IL-1, IL-4 and IL-10 secretion. Additionally, regulatory T cells (Treg) play a vital role in the immune system by inhibiting and reducing the differentiation of Th cells. Th1 cells activate resting macrophages (M) through the secretion of IFN-gamma and TNF-alpha and migrate from lymph nodes to the central nervous system (CNS) through the blood-brain-barrier (BBB) under the influence of IL-12 and specific chemokines. Th2 cells play a crucial role in activating naive B-cell under the presence of IL-4, IL-5, IL-6, IL-10 and IL-13. Active B-cells may then duplicate and differentiate into plasma B-cells that release class G immunoglobulins (IgG) targeting MBP and MBP-presenting cells, which can leave the lymph node compartment and move into the CNS.

Th17 cells secrete various cytokines, including IL-17, IL-6, IL-21, IL-22, IL-23, and TNF-α. Subsequently, these cells migrate across the blood-brain barrier (BBB) to the central nervous system (CNS) under the influence of specific cytokines, namely IL-17, IL-6, and IL-22, as well as IL-23, provided that the inhibitory effects of IL-10 are absent. According to the role of Il-10, which leads to the suppression of antigen-specific proliferation and inhibition of the synthesis of Th cells-related cytokines (IFN-γ, TNF-α, TNF-β, IL-1, IL-2, IL-6), the migration of Th1, activated macrophages, Th17 and IgG can be affected. MHC-I expression and presentation are essential for CD8^+^ T-cells to activate cytotoxic and Treg functions, while active CD8^+^ T cells leave the lymph node and move to CNS. Tregs have a significant role in mitigating inflammatory processes by releasing IL-10. Also, the BBB is compromised and damaged by the interaction of specific cytokines secreted by T-cells [30].

On the other hand, Epstein-Barr virus (EBV) has been implicated in the development of MS through a mechanism known as "molecular mimicry " This occurs when the surface molecules of EBV resemble host CNS antigens, specifically myelin proteins, resulting in the immune system's activation against the host's tissues. EBV can activate both B and T cells through molecular mimicry, which entails several steps. Firstly, EBV attaches to specific receptors on the surface of naive B cells, initiating a signaling pathway that leads to the proliferation and differentiation of B cells into plasma cells that synthesize antibodies against EBV. The activation of T cells by EBV is also a multi-step process involving the recognition of EBV antigens presented by infected B cells, activated DC and the activation of intracellular signaling pathways, which lead to the differentiation and proliferation of T cells [31]. In the CNS, activated helper T cells engage with resident microglia. Upon recognition of myelin antigens, the microglia are reactivated and produce specific cytokines (such as IL-1, IL-6, IL-12, TNF-α, IFN-γ, and IL-2) and chemokines that activate Th1 and Th17 cells, leading to an inflammatory cascade and subsequent destruction of oligodendrocytes. Th17 cells, via secretion of IL-17, recruit neutrophils from the bloodstream into the CNS. Additionally, reactivated astrocytes release pro-inflammatory cytokines, while IgG, CD8 + cells, and macrophages, under the influence of specific cytokines, can attack oligodendrocytes. Furthermore, the presence of plasma B cells that generate cross-reactive antibodies against myelin antigens and memory B cells that serve as antigen-presenting cells for the cross-reactive T cells can stimulate the production of pro-inflammatory cytokines. This process may initiate the immunopathological cascade observed in multiple sclerosis (MS), ultimately resulting in myelin damage [32].

Furthermore, secondary activation of T and B cells inside the CNS is also considered. The computational model implements the blood-brain barrier compartment (BBB). As a result, we simulated the possible damage of BBB due to local inflammation. In this case, T and B cells, even if not activated, can penetrate damaged BBB and a CNS-located secondary activation may happen.

### 2.2 MS treatment conceptual model

In order to accurately simulate the impact of cladribine and ocrelizumab on the immune system and develop the treatment layers in UISS-MS, we obtained precise information regarding their mechanisms of action from relevant and specialized literature sources [33], and we developed the treatment conceptual model as a final step.

**Fig. 2:**
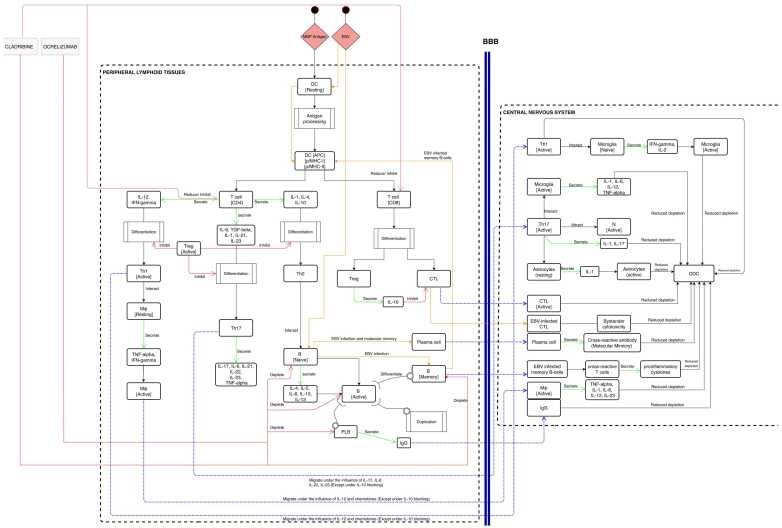
MS treatment conceptual model. Schematic description of the main immune system entities and interactions related to MS. The central nervous system and the peripheral lymphoid tissues, the two major compartments, are shown. Moreover, the mechanism of action of cladribine and ocrelizumab is inserted.

Cladribine treatment is one of the most recently approved MS medicine. The mechanism of action of cladribine results in profound, rapid and long-lasting reductions of CD4^+^ and CD8^+^ T cells, as well as B cells, each in their magnitude and kinetics [17], [34], [35]. Cladribine is widely recognized as a T and B cell inhibitor, particularly emphasizing its T cell inhibitory effects as the primary mechanism of action. Nevertheless, immunophenotyping data has indicated that effective oral doses of cladribine induce only a modest 20–30 % depletion of CD8 + T cells and a 40–45 % depletion of CD4 + T cells within a 12-month. In contrast, cladribine elicited a notable depletion of CD19 + B cells, reaching 80–85 % depletion. Ocrelizumab is a humanized monoclonal antibody of recombinant origin that specifically targets CD20 expression on B cells, affecting pre-B, naive, active and memory B-cells by depleting them. This depletion of B cells reduces the number of immune cells that attack myelin and cause inflammation in MS patients. Moreover, ocrelizumab may modulate T cells by modifying their activation and function [14] (Fig. 2).

## 3 Results

UISS-MS was able to predict the expected effects of cladribine and ocrelizumab over virtual MS patients that were digitally created using the same demographic characteristics and the lesional load of the one enrolled in the clinical trials we used as a reference for validation purposes.

The digital patient generation needs to be fed with different inputs that are biologically and physiologically plausible. Simulator parameters were set based on the characteristics of the patients at baseline (age, sex, weight, race) enrolled into the “Clarity” and “Clarity extension” randomized controlled trials (RCT) [24], [25]. In order to account for the considerable heterogeneity of predicted MS dynamics resulting from the aforementioned baseline features, we randomly varied the MHC-I major antigens (HLA A, B and C) and the MHC-II locus (DM, DO, DP, DQ and DR) and ran the simulator using multiple different immune system repertoires for each digital patient. Therefore, thousands of different profiles were generated. Finally, 3000 digital patients that matched the behaviors reported in the "Clarity" study regarding the frequency of relapses and lymphocytopenia adverse reactions were selected. The generated cohort has been used to validate the model also in the "Clarity- extension" study [36], a two-year extension study in which placebo recipients from “clarity” received cladribine 3.5 mg/kg, while cladribine recipients were re-randomized 2:1 to cladribine 3.5 mg/kg or placebo, with blind maintained. The UISS-MS can detect a variety of forms of “relapsing activity.” The first is related to a slight oligodendrocyte loss that indicates a biological event not detectable from MRI. Other forms are related to a consistent oligodendrocytes loss that instead provokes an MRI-detectable lesion. Only relapses that are detectable by MRI were considered in our analysis. UISS-MS simulates three cubic millimeters of white matter with 50 × 10^3^ ODC per cubic millimeter (equivalent to 150 ×10^3^ ODC) to estimate the ODC loss and any related MRI-detectable event. The minimum MRI detectable threshold is set as a lesion involving at least 50 × 10^3^ ODC, which assumes a uniform distribution of ODCs in the white matter tissue. Grahl et al. work [36] was referred to establish this threshold. If the ODC loss is equal to or greater than 50 × 10^3^, it indicates the presence of one or more lesions that MRI can detect.

We also used Levene's test for the 3000 selected digital patients. In inferential statistics, Levene's test addresses data drawn from non-normal distributions and is a robust tool to check the homogeneity of variances [37]. According to Levene's test, we applied the null hypothesis (the so-called $H_{0})$ to check whether there is a correlation among patient groups.

The same procedure for cladribine was used to design real patients’ simulations for ocrelizumab. First, we set the simulator parameters based on the demographic characteristics of the patients at baseline (age, sex, race, time since symptom onset and diagnosis, and number of relapses in the previous 12 months) enrolled in the “Opera I” and “Opera II” RCT [23]. Then, for each virtual patient, we ran the simulator using multiple different immune system repertoires, randomly varying the MHC-I major antigens (HLA A, B and C) and MHC-II locus (DM, DO, DP, DQ and DR) to take into account the Opera studies distribution (i.e., about 25 % USA population and the remaining from the rest of world). Thus, we generated different thousands of profiles and we finally selected 200 digital patients (100 for Opera I and 100 for Opera II) that match the behavior (i.e., in terms of Annualized Relapse Rate (ARR) in participants with relapsing multiple sclerosis at 96 weeks) as reported in the abovementioned study. In our in silico study, we calculated ARR the same way the Opera trials did, using a negative binomial model.

Furthermore, we complied with recently published information detailing the best technique to compute ARR using a negative binomial model to prevent errors [38]. A robust methodology we recently validated and published was used to augment the in silico cohorts at 1000 digital patients for Opera I in silico trial and 1000 digital patients for Opera II in silico trial [39]. As for cladribine, we considered only relapses that are MRI detectable and used the same method we described for cladribine to assess if a relapse is MRI detectable.

For the cladribine treatment, to be sure that all samples owned equal variances, Levene's test was used, as mentioned before. Levene’s statistical test assumes that although different samples can come from populations with different means, they have the same variance to be treated like a homogeneous group. If the variance from the samples is statistically the same or more generally similar, it is possible to count each patient as independent and mix all patients.

Since the *statistic* score is close to 1 and the *p_value* is ≥0.05 (as shown in Table 1), we cannot reject the null hypothesis (the so-called $H_{0})$, so we can confirm that all digital patients have equal variances.

**Table 1:** Levene’s test result for 3000 selected digital patients treated with cladribine.

Data	Statistic	p_value
All profiles (3000 DP)	0.9180	0.9389

Concerning the analysis of variance among replicates, we obtained that we cannot reject the null hypothesis $H_{0}$ for the whole set of replicates. If the variance from the replicates is statistically the same or more generally similar, it is possible to count each patient as independent and mix all patients. This means that replicates can also be treated as one single group.

Table 2, Table 3 show the results of simulations with the digital patient profiles that best match the data retrieved from the “Clarity” study.

**Table 2:** Number of relapses in simulated “clarity” RCT at week 96.

Number of relapses	Placebo (N = 1000)	cladribine 3.5 mg/kg (N = 1000)	cladribine 5.25 mg/kg (N = 1000)
0	605	800	790
1	250	160	170
2	98	30	29
3	40	10	10
>= 4	7	0	1

**Table 3:** Lymphocytopenia adverse events in simulated “clarity” RCT.

Grade (*)	Placebo N = 1000 (%)	cladribine 3.5 mg/kg N = 1000 (%)	cladribine 5.25 mg/kg N = 1000 (%)
0	83.73	19.9	3.9
1	9.87	25.87	13.59
2	6.4	31.4	41.4
3	0	21.82	38.10
4	0	1.01	3.01

(*) grade 1: absolute lymphocytes count (ALC) 800–999/μl; grade 2: ALC 500–799/μl; grade 3: ALC 200–499/μl; and grade 4: ALC< 200/μl [40].

Looking at Table 2, Table 3, one can observe that both the number of relapses and the lymphocytopenia adverse events in simulated “clarity” RCT mirror the ones reported in the “clarity” study [25].

Table 4, Table 5, Table 6, Table 7 show the results of UISS-MS in predicting the outcomes of efficacy and adverse reactions in the “clarity-extension” study [26]. We ran the simulations on two different randomly generated cohorts. It is worth mentioning that we used the same digital cohort selected for the calibration in the “clarity” study. This validates the cladribine extension of UISS-MS retrospectively.

**Table 4:** Number of relapses in simulated “clarity extension” – sub-cohort a – RCT at month 42.

Relapse free patients	CP 3.5 mg/kg (n = 300)	CP 5.25 mg/kg (n = 300)	CC 7 mg/kg (n = 300)	CC 8.75 mg/kg (n = 300)	PC 3.5 mg/kg (n = 300)
	225	226	243	231	240

**Table 5:** Number of relapses in simulated “clarity extension” – sub-cohort b – RCT at month 42.

Relapse free patients	CP 3.5 mg/kg (n = 300)	CP 5.25 mg/kg (n = 300)	CC 7 mg/kg (n = 300)	CC 8.75 mg/kg (n = 300)	PC 3.5 mg/kg (n = 300)
	219	220	238	225	246

**Table 6:** Lymphocytopenia adverse events in simulated “clarity extension” – sub-cohort a – RCT.

Grade (*)	CP 3.5 mg/kg (n = 300)	CP 5.25 mg/kg (n = 300)	CC 7 mg/kg (n = 300)	CC 8.75 mg/kg (n = 300)	PC 3.5 mg/kg (n = 300)
1					
2					
3	16	20	115	150	73
4	0	0	8	10	2

(*) grade 1: absolute lymphocytes count (ALC) 800–999/μl; grade 2: ALC 500–799/μl; grade 3: ALC 200–499/μl; and grade 4: ALC< 200/μl [40].

**Table 7:** Lymphocytopenia adverse events in simulated “clarity extension” – sub-cohort b – RCT.

Grade (*)	CP 3.5 mg/kg (n = 300)	CP 5.25 mg/kg (n = 300)	CC 7 mg/kg (n = 300)	CC 8.75 mg/kg (n = 300)	PC 3.5 mg/kg (n = 300)
1					
2					
3	15	21	117	148	71
4	0	0	9	9	3

(*) grade 1: absolute lymphocytes count (ALC) 800–999/μl; grade 2: ALC 500–799/μl; grade 3: ALC 200–499/μl; and grade 4: ALC< 200/μl [40].

The treatment groups can be summarized as follows:

• CP 3.5 mg/kg means cladribine tablets 3.5 mg/kg in CLARITY/placebo in CLARITY Extension;
• CP 5.25 mg/kg means cladribine tablets 5.25 mg/kg in CLARITY/placebo in CLARITY Extension;
• CC 7 mg/kg means cladribine tablets 3.5 mg/kg in CLARITY/cladribine tablets 3.5 mg/kg in CLARITY Extension;
• CC 8.75 mg/kg means cladribine tablets 5.25 mg/kg in CLARITY/cladribine tablets 3.5 mg/kg CLARITY Extension;
• PC 3.5 mg/kg means placebo in CLARITY/cladribine tablets 3.5 mg/kg in CLARITY Extension.

For the ocrelizumab treatment, as for the cladribine one, Levene's test was used to be sure that all samples owned equal variances. The obtained results are shown in Table 8.

**Table 8:** Levene’s test result for 3000 selected digital patients treated with ocrelizumab.

Data	Statistic	p_value
All profiles (2000 DP)	0.9243	0.9569

Since the *statistic* score is close to 1 and the *p_value* is ≥0.05, we cannot reject the null hypothesis $H_{0}$, so we can confirm that all digital patients have equal variances.

Concerning the analysis of variance among replicates, we obtained that we cannot reject the null hypothesis $H_{0}$ for the whole set of replicates. If the variance from the replicates is statistically the same or more generally similar, it is possible to count each patient as an independent one and to mix all patients. This means that replicates can also be treated as one single group.

We simulated the two arms of treatment matching the RCT: IFNβ− 1a 44 mcg SC injections three times per week for the first arm and ocrelizumab 600 mg intravenous (IV) as 300 mg infusions on Days 1 and 15 for the first dose and as a single infusion of 600 mg for all subsequent infusions every 24 weeks. The simulation time was set to 100 weeks.

The following tables are the results of simulations with the digital patient profiles that best match the data retrieved from the Opera I and II studies. (Table 9).

**Table 9:** ARR at 96 weeks in “Opera I and II” in silico trials.

Arm	Number of Simulated Digital Patients	ARR at 96 weeks in Opera I	ARR at 96 weeks in Opera II
IFNβ− 1a	500 (Opera I) + 500 (Opera II)	0.352 (from 0.291 to 0.437)	0.346 (from 0.275 to 0.430)
ocrelizumab	500 (Opera I) + 500 (Opera II)	0.195 (from 0.146 to 0.243)	0.191 (from 0.143 to 0.239)

As one can envisage from the analysis of Table 1, the ARR among patients receiving ocrelizumab at 96 weeks is predicted to be 0.193 against real Opera RCT of 0.16.

ARR IFNβ− 1a is predicted to be 0.349, compared with real Opera RCT 0.29.

The in silico trial of the Opera study predicted a relative reduction of 45.7 % on ocrelizumab compared to IFNβ− 1a. This is in excellent agreement with the 50 % relative reduction predicted in the real RCT, given that the digital patients can own more variability than those enrolled in the Opera RCT.

**Fig. 3:**
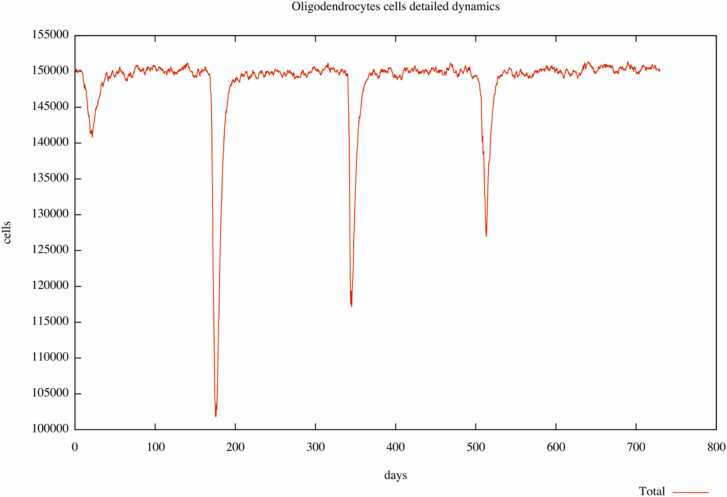
Digital patient U001 without any treatment. Three relapses are highlighted during the observational time of two years.

**Fig. 4:**
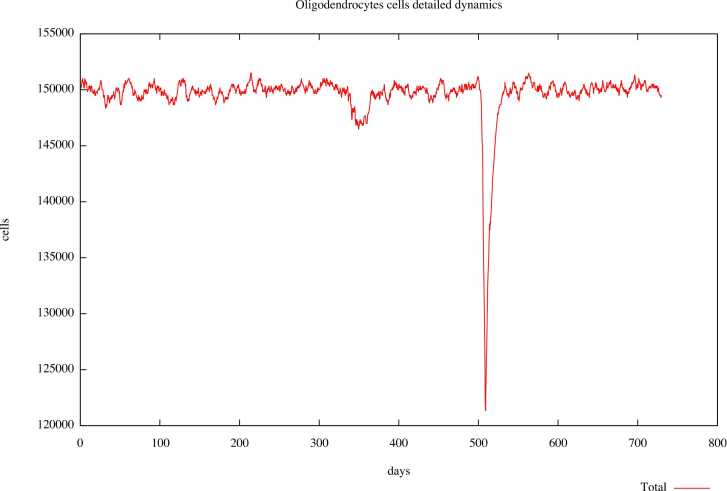
Digital patient U001 was treated with ocrelizumab. One relapse is highlighted during the observational time of two years.

The plots in Figs. 3 and 4 depict the relapses during the observational time (two years) in an untreated digital patient and the same patient treated with ocrelizumab.

The plots represent the number of oligodendrocytes cells during time expressed in days, and relapse is shown as a rapid reduction in cell number. For example, in the untreated digital patient (Fig. 3), during the observational time, we can observe three relapses represented as three peaks with a downward trend, meaning a loss of oligodendrocytes at those times. Instead, in the plot describing the dynamics in the same subject treated with ocrelizumab (Fig. 4), we observe only one peak, which tends downwards, meaning only one relapse. The reduction in the number of relapses in pharmacologically treated digital patients allows us to confirm the capability of UISS-MS in predicting the activity of the ocrelizumab in terms of its ability to reduce the loss of oligodendrocytes during MS disease.

## 4 Discussion

MS is an autoimmune disease characterized by chronic inflammation of the CNS with myelin and axonal loss, leading to severe disability [32]. Its clinical course may vary, but the relapsing-remitting form is the most common clinical phenotype [41], [42]. Therefore, the mainstay of MS treatment is immunomodulatory/immunosuppressant drugs employed to control inflammation and prevent relapses [43]. Among them, cladribine and ocrelizumab are two high-efficacy treatments currently used for patients with elevated disease activity. Their mechanism of action involves both B and T cells, and they have been demonstrated to be highly effective in reducing the number of relapses in RRMS [44].

Nowadays, the so-called “in silico trials” could help design real randomized clinical trials, covering every single stage, from phase I to post-market surveillance. In silico trials use computer modeling and simulation platforms to reproduce the dynamics of the intended context of use (physiology, disease, treatment) over a population of virtual humans,^2^ to predict the effect of a treatment on a disease or to help clinicians in deciding the best therapeutic strategy based on an individualized patient profile. UISS-MS is a mechanistic in silico trial platform specifically designed to reproduce the intricate dynamics of multiple sclerosis - human immune system interaction. UISS-MS already owned a treatment library containing different drugs used against the evolution of MS. In this study, we designed two new treatment layers over UISS-MS to simulate and validate the effects of cladribine and ocrelizumab treatments in reducing relapse rates in patients with RRMS. For cladribine layer validation, we generated 3000 digital patients according to the patients' features in the “Clarity study.” This cohort has also been used to validate the model in the “Clarity-extension” study and to predict the drug’s efficacy. Moreover, we highlight the UISS-MS capability to predict the side effects affecting the lymphocytes induced by cladribine, differentiated in severity grades.

While for the ocrelizumab layer, we generated 200 digital patients based on the data of patients included in Opera trials (100 for Opera I and 100 for Opera II) and calculated their ARR. Then, we extended the in silico cohorts to 1000 digital patients for Opera I in silico trial and 1000 digital patients for Opera II in silico trial. In silico results mirrored the “Clarity” and “Opera” clinical trials outcomes. Moreover, cladribine and ocrelizumab effects on relapses were correctly captured by UISS-MS, analyzing the effect in reducing the loss of oligodendrocytes induced by the two drugs under investigation.

Similarly, we conducted an in silico trial for ocrelizumab. Results over the digital patients treated with ocrelizumab showed a measurable decrease in the frequency of relapses. Through this retrospective validation of a new treatment layer, we further refined the UISS-MS platform.

Potentially, UISS-MS can generate subject-specific predictions, i.e., the expected accuracy is that the predicted value is sufficiently close to the value measured experimentally in each individual in the reference population. A specific individualized patient profile should be available to obtain such a level of prediction. However, this study's only available data is at a population level (demographics information). Nevertheless, in this worst-case scenario, we can still capture a subpopulation that matches the desired statistical profile.

Overall, UISS-MS can provide evidence to predict the effects of MS modifying therapies over the relapses activity and reveal eventual adverse consequences.

## 5 Conclusions

In order to manage human health effectively, decision-makers need to make informed choices that can impact individuals or groups of people (referred to as the reference population). Examples include clinicians deciding on personalized treatments, researchers selecting druggable targets in biomedical research, healthcare managers planning policies, and biomedical companies striving to minimize animal and human experimentation for regulatory approval of new products.

In Silico Medicine, which encompasses the use of modeling and simulation technologies in healthcare, can be categorized into three main types based on the user: Digital Patient solutions, which employ models' predictions for clinical decision support systems; In Silico Trials solutions, which use computer models to assess the safety and effectiveness of new medical products; and Personal Health Forecasting solutions, where patients themselves are the end users.

UISS-MS has a long development history: it belongs to both In Silico Trials and Digital patient solutions as it can represent the disease dynamics and the response of several treatments, both for an individual and an average cohort of digital patients. With this last effort, we enlarged the library of treatment layers, intending to make UISS-MS ready to assist MS specialists in predicting the course of the disease, formulating an early prognosis, and selecting a personalized treatment strategy. Furthermore, pharmaceutical companies and researchers can take advantage of designing new therapies using UISS-MS as an in silico trial platform to envisage the effects of the newly developed treatment on MS patients.

However, before utilizing emerging technologies to support human experimentation in drug development and new market authorization submissions like UISS-MS, it is crucial to adequately address the assessment of the model's credibility, despite the acknowledged value of these technologies.

Verification, validation, uncertainty quantification (VVUQ), and applicability assessment establish a computational model's credibility. However, precise regulatory pathways are still missing. FDA relies on ASME VV40^3^ to assess in silico trials applied in medicinal products, while EMA is considering these new approaches through its “Regulatory Science Strategy to 2025”.^4^ Qualification pieces of advice are one of the fundamental instruments to qualify in silico trials for regulatory approval. We are now proceeding to assess the UISS-MS credibility following ASME VV40 rules and this, if completed, would represent a milestone in widening the usage of in silico trials and digital patients.

## CRediT authorship contribution statement

**Avisa Maleki** and **Elena Crispino:** Validation, Data curation, Writing – original draft, Writing – review & editing. **Serena Italia:** Writing – review & editing. **Valentina Di Salvatore:** Writing – review & editing. **Maria Assunta Chiacchio:** Writing – review & editing. **Fianne Sips** and **Roberta Bursi:** Writing – review & editing. **Giulia Russo:** Writing – review & editing, Formal analysis. **Davide Maimone:** Writing – review & editing. **Francesco Pappalardo:** Conceptualization, Supervision, Methodology, Software, Writing – original draft.

## Declaration of Competing Interest

The authors declare no conflict of interest.

## Footnotes

2 https://doi.org/10.48550/arXiv.2304.06678.

3 https://www.asme.org/codes-standards/find-codes-standards/v-v-40-assessing-credibility-computational-modeling-verification-validation-application-medical-devices.

4 https://www.ema.europa.eu/en/about-us/how-we-work/regulatory-science-strategy.
